# Comparison of rumen bacterial communities between yaks (*Bos grunniens*) and Qaidam cattle (*Bos taurus*) fed a low protein diet with different energy levels

**DOI:** 10.3389/fmicb.2022.982338

**Published:** 2022-09-06

**Authors:** Hu Liu, Tao Ran, Chengfu Zhang, Wenzhu Yang, Xiukun Wu, Allan Degen, Ruijun Long, Zunji Shi, Jianwei Zhou

**Affiliations:** ^1^State Key Laboratory of Grassland Agro-Ecosystems, Center for Grassland Microbiome, College of Pastoral Agriculture Science and Technology, Lanzhou University, Lanzhou, China; ^2^International Centre for Tibetan Plateau Ecosystem Management, College of Ecology, Lanzhou University, Lanzhou, China; ^3^State Key Laboratory of Barley and Yak Germplasm Resources and Genetic Improvement, Institute of Animal Science and Veterinary, Tibet Academy of Agricultural and Animal Husbandry Sciences, Lhasa, China; ^4^Lethbridge Research and Development Centre, Agriculture and Agri-Food Canada (AAFC), Lethbridge, AB, Canada; ^5^Northwest Institute of Ecological Environment and Resources, Chinese Academy of Sciences, Lanzhou, China; ^6^Desert Animal Adaptations and Husbandry, Wyler Department of Dryland Agriculture, Blaustein Institutes for Desert Research, Ben-Gurion University of the Negev, Be’er Sheva, Israel

**Keywords:** yaks, rumen bacterial community, Qaidam cattle, low protein diet, energy levels

## Abstract

The yak (*Bos grunniens*), an indigenous bovine on the Qinghai-Tibetan plateau (QTP), is reported to digest low quality forage to a greater extent and to require less protein and energy for maintenance than the introduced Qaidam cattle (*Bos taurus*). Ruminal bacteria play a major role in feed degradation, and therefore, we hypothesized that ruminal bacteria composition would differ between yaks and cattle, and confer an advantage to yaks for poor quality diets. To test our hypothesis, we determined the ruminal bacteria profiles, rumen fermentation parameters, and enzyme activities in these bovine species consuming a low-protein diet differing in energy level. Six castrated yaks (155 ± 5.8 kg) and 6 castrated Qaidam cattle (154 ± 8.0 kg) were used in two concurrent 4 × 4 Latin square designs with 2 additional animals of each species in each period. The animals were offered a low-protein diet of 70.4 g/kg dry matter (DM) and one of four metabolizable energy levels, namely 6.62, 8.02, 9.42, and 10.80 MJ/kg. Ruminal pH, concentrations of ammonia-N and total volatile fatty acids (VFAs), the molar proportion of acetate, and the ratio of acetate to propionate (A:P) were greater (*P* < 0.05), whereas the molar proportion of propionate was lesser (*P* = 0.043) in yaks than in cattle. With increasing dietary energy level, ruminal pH, the molar proportion of acetate and the ratio of A:P decreased linearly (*P* < 0.05), whereas, the concentration of total VFAs, molar proportions of propionate, butyrate, iso-butyrate, and iso-valerate and concentration of ammonia-N increased linearly (*P* < 0.05). The relative abundance (RA) of Firmicutes increased linearly (*P* < 0.01), whereas, the RA of Bacteroidetes decreased linearly (*P* < 0.001) with increasing energy level in both bovine species. The RAs of *Prevotella* and *Rikenellaceae_RC9_gut_group* decreased linearly (*P* < 0.05) with increasing energy level in both yaks and cattle. The RAs of fibrolytic (e.g., *Rikenellaceae_RC9_gut_group*), and H_2_-incorporating (e.g., *Quinella*) bacteria were greater (*P* < 0.05) in yaks than in cattle. We concluded that the two bovines differ in ruminal bacterial profiles and rumen fermentation parameters, and confer an advantage to yaks over cattle in consuming a low protein diet with differing energy level.

## Introduction

The high-altitude Qinghai-Tibetan Plateau (QTP), known as “the roof of the world,” originated about 40 million years ago. The plateau is characterized by prolonged cold weather, strong ultraviolet radiation, heavy winds, and low air oxygen content. There is a short forage growing season of 120 days, with very sparse pasture of poor quality over the long winter. Yaks (*Bos grunniens*) were domesticated by the ancient Qiang people approximately 7300 years ago (Qiu et al., 2012), and are well-adapted to the extreme conditions of the QTP. There are currently more than 16 million domestic yaks worldwide, of which 15.2 million are raised on the QTP (Zhang et al., 2015). The yak has been closely linked with human civilization and daily life, providing meat, milk, leather, and dung, as well as transportation. Qaidam cattle (*Bos taurus*) were introduced to the QTP by Tibetans approximately 1700 years ago (China National Commission of Animal Genetic Resources, 2011; Chen et al., 2018), and the current population is close to 10,000. The yaks graze alpine grassland at altitudes between 3,000 and 6,000 m above sea level (a. s. l.) all year round without supplements. During the long, cold winter, yaks mobilize their body reserves as energy intake is insufficient, and lose substantial body weight. Cattle are raised in agro-pastoral transition zones at altitudes between 2,600 and 3,600 m a. s. l. and require supplements and shelter at night in winter. The two bovine species co-graze over their overlapping ranges.

Although yaks and Qaidam cattle are both capable of subsisting on high-fiber diets, it has been reported that yaks are less discriminate herbivores, digest fiber to a greater extent, and produce more volatile fatty acids (VFAs) and microbial protein than cattle when fed the same diet (Zhou et al., 2018; Liu et al., 2022). These differences between bovine species suggest they differ in their rumen microbial communities (Huang et al., 2012; Zhang et al., 2016; Huang X. D. et al., 2021), that enable yaks to cope with a low-quality diet better than cattle. It was reported that the CAZymes (degradation carbodydrates) and fibrolytic activities were greater in yaks than in cattle (Guo, 2021). Yaks also had greater relative abundances (RAs) of Thermoplamatales-affiliated Linage C (TALC), *Christensenellaceae_R7_group*, and *Lachnospiraceae_UCG_008 group* but lesser RAs of *Prevotella* and *Succiniclasticum* than cattle (Huang et al., 2012; Xin et al., 2019). We hypothesized that the ruminal bacterial profiles and fermentation parameters would differ between the two bovine species when fed different quality diets. To test this hypothesis, we determined the rumen bacterial profiles, and rumen fermentation parameters of yaks and cattle when fed a low protein diet differing in energy levels.

## Materials and methods

This experiment was carried out at Wushaoling Yak Research Facility of Lanzhou University (102°51.7’E, 37°12.4’N, altitude 3,154 m. a. s. l.), situated in the northeastern part of the QTP, Tianzhu Tibetan Autonomous County, Wuwei City, Gansu Province, China.

### Animals, design, and diets

Six yaks (155 ± 5.8 kg) and 6 Qaidam cattle (154 ± 8.0 kg), all castrated males and 2.5 years of age, were held individually in metabolic cages (2.2 m × 1.0 m). The experiment was a 2 (genotypes) × 4 (energy levels) factorial arrangements and conducted as two concurrent 4 × 4 Latin square designs balanced for carry-over effects, with 2 additional yaks and cattle in each period. Treatment sequences for the two additional animals of each species were selected randomly from columns of a separate Latin square (Bailey et al., 2012). In each period of 28 days, 4 yaks and 4 cattle received one of 4 diets differing in energy level, and the two additional animals of each species received one of the four diets (Sarraseca et al., 1998). The six animals of each species consumed all four dietary treatments.

To ensure each animal consumed all the feed offered daily, the least voluntary intake was determined before the study and offered throughout the feeding trial. Therefore, 2.75 kg/d DM per animal of pellets (total mixed ration; Gansu Runmu Biological Engineering Co., Ltd, Jinchang, China) were offered in equal portions at 08:00 and 18:00 with water freely available. The diets were formulated to be isonitrogenous with low crude protein (CP, 74 g/kg DM), but with incremental levels of metabolizable energy (ME): 6.62, 8.02, 9.42, and 10.80 MJ/kg (Table 1; 14). The dietary CP content was lower than the recommendation of NRC (National Research Council, 2000) for beef cattle, but was similar to the average CP content of forage in on the QTP during the cold season (Xie et al., 1996). The diets provided 0.8, 0.9, 1.0, and 1.1 times maintenance energy requirement of growing beef cattle according to NRC (National Research Council, 2000).

**TABLE 1:** Ingredients and chemical composition of experimental diets.

Items	Dietary ME level, MJ/kg DM			
	
	6.62	8.02	9.42	10.80
**Ingredient, g/kg DM**				
Corn straw^1^	850	700	550	400
Corn grain, ground^2^	46.0	117	180	260
Corn husk	11.0	55.0	110	154
Cotton meal	27.0	24.3	21.0	18.5
Soybean meal	27.0	24.0	21.0	18.0
Wheat bran	24.0	21.0	16.0	7.00
Corn starch	–	40.0	72.0	104
Rumen bypass palm oil	–	4.00	13.0	20.5
Calcium hydrophosphate	–	–	2.00	3.00
Sodium chloride	10.0	10.0	10.0	10.0
Commercial premix^3^	5.00	5.00	5.00	5.00
**Chemical compositions, g/kg DM**				
DM, g/kg as-fed	973	969	966	934
OM	864	885	902	901
CP^4^	74.5	74.4	74.4	74.3
aNDF	604	544	486	422
ADF	322	276	232	186
Ether extract	55.7	66.8	80.3	91.6
Calcium	8.70	8.50	7.90	7.00
Phosphorus	2.40	2.30	2.40	2.40
Gross energy, MJ/kg	16.0	16.4	16.9	17.2
ME, MJ/kg^5^	6.62	8.02	9.42	10.80

DM, dry matter; OM, organic matter; CP, crude protein; ME, metabolizable energy; aNDF, neutral detergent fiber; ADF, acid detergent fiber.

^1^The DM, CP, EE, NDF, ADF, and Ash of corn straw were 800, 50, 12, 700, 440, and 70 g/kg DM, respectively.

^2^DM, CP, EE, NDF, ADF, and Ash of corn grain were 860, 80, 36, 99, 31, and 12 g/kg DM, respectively.

^3^The premix provided the following per kg of premix: Vitamin A 3 000 000 IU, Vitamin D 375 000 IU, Vitamin E 220 IU, biotin 12 mg, lysine 13 000 mg, Cu (as copper sulfate) 1 200 mg, Fe (as ferrous sulfate) 3 000 mg, Mn (as manganese sulfate) 2 000 mg, Zn (as zinc sulfate) 4 000 mg, I (as potassium iodide) 15 mg, Se (as sodium selenite) 20 mg.

^4^CP calculated as 6.25 × N content.

^5^The ME was calculated according to the Tables of Feed Composition and Nutritive Values in China (Xiong et al., 2018).

### Procedures and sample collection

In each period, approximately 100 g of each diet were collected daily from d 23 to 28. Two h after morning feed was offered on d 28, an oral stomach tube (Anscitech Co. Ltd., Wuhan, China) was used to collect 150 mL of rumen fluid. The first 50 mL of fluid were discarded to avoid saliva contamination. The pH of the rumen fluid was measured immediately using a pH meter (PB-10, Sartorius Co., Göttingen, Germany), and then the fluid was filtered through 4 layers of cheesecloth. Five mL of filtrate were mixed with 5 mL of deproteinizing solution (100 g metaphosphoric acid and 0.6 g crotonic acid per liter) for VFAs analysis; 5 mL of filtrate were mixed with 5 mL hydrochloric acid solution (0.5 mmol/L) for ammonia N measurement; and the rest was stored at −80°C for analyzing bacterial community and enzyme the activity.

### Feed analysis

Feed samples were dried at 65°C in a forced air oven for 72 h, and ground through a 1-mm sieve (JFSO-100, Topu Yunnong Instrument, Hangzhou, China). The DM (method 925.45), organic matter (method 942.05), and ether extract (method 920.29) were determined according to the Association of Official Analytical Chemists (AOAC, AOAC, 2006). Total nitrogen content was determined using a nitrogen analyzer (K1000, Hannon Instruments, Jinan, China), and CP was calculated as total N × 6.25. Neutral detergent fiber (aNDF) and acid detergent fiber (ADF) were determined by an automatic fiber analyzer (Ankom Technology, Fairport, NY, United States) according to Robertson and Van Soest (1981) and Van Soest et al. (1991), respectively. Ten g of sodium sulfate per aNDF solution and 0.5 g heat-stable α-amylase per sample were added to the aNDF solution. The ADF was determined on the residue of the aNDF. Gross energy of feed was determined by bomb calorimetry (6400 Calorimeter, Parr Instrument Company, Moline, Illinois, United States).

### Rumen fermentation parameters

The VFAs concentration of rumen fluids was determined by gas chromatography (GC) with a capillary column (AT-FFAP: 30 m × 0.32 mm × 0.5 μm) using a Shimadzu 2010 plus system (Shimadzu Corporation, Kyoto, Japan) following Liu et al. (2021b). Ammonia-N concentration was analyzed using a spectrometer (SpectraMax M5, Molecular Devices, San Jose, United States) at an absorbance of 630 nm, following Hristov et al. (2001).

Ruminal enzyme activities of carboxymethylcellulase (CMCase), amylase, xylanase, and pectinase were measured by commercial enzyme-linked immune sorbent assay (ELISA) kits (product No: BYE98162, BYE98329, BYE98332, and BYE99021, respectively; Shanghai Bangyi Biological Technology Co. Ltd., Shanghai, China). The rumen fluid was centrifuged at 800 × g for 5 min at 4°C, and the supernatant was shaken ultrasonically for 3 min. The enzyme activities were determined using a spectrometer (SpectraMax M5, Molecular Devices, San Jose, United States) at an absorbance of 450 nm.

### DNA extraction, 16S rRNA gene amplification, and sequencing

After the rumen fluid was thawed on ice, total genomic DNA of rumen bacteria was extracted from 1 mL by using the E.Z.N.A^®^ kit (Omega Bio-tek, Norcross, GA, United States), and following the manufacturer’s instructions. The concentration and the purity of the extracted DNA were determined by the 260/280 nm ratio (1.8 to 2.2) using a NanoDrop 2000 UV-vis Spectrophotometer, (Thermo Scientific, Wilmington, DE, United States). The quality of the extracted DNA was tested using 1% agarose gel electrophoresis (Axygen Biosciences, Union City, CA, United States). The extracted DNA samples were stored in sterile centrifuge tubes (Thomas Scientific, Wilmington, DE, United States) at –80°C for later analysis.

The conventional polymerase chain reaction (PCR) amplification and bioinformatic analysis of extracted DNA samples were done by Shanghai Majorbio Bio-Pharm Technology Co., Ltd (Shanghai, China). The hypervariable V3-V4 region of the bacterial 16S rDNA gene was amplified using primers pair 338F (5′-ACTCCTACGGGAGGCAGCAG-3′) and 806R (5′-GGACTAC HVGGGTWTCTAAT-3′). The bacterial 16S amplification and the quality-filter, cluster, and analysis of 16S rRNA sequencing data followed Liu et al. (2019). The reaction conditions and procedures of PCR amplification of the 16S rRNA gene were as follows: initial denaturation at 95°C for 3 min, followed by 27 cycles of denaturing at 95°C for 30 s, annealing at 55°C for 30 s and extension at 72°C for 45 s, and a final single extension at 72°C for 10 min. The PCR mixtures were prepared in triplicate 20 μL volumes which consisted of 4 μL of 5 × TransStart FastPfu buffer, 2 μL of 2.5 mM deoxyribonucleotides triphosphate (dNTPs), 0.8 μL of forward primer (5 μM), 0.8 μL of reverse primer (5 μM), 0.4 μL of TransStart FastPfu DNA Polymerase, 10 ng of template DNA, and ddH_2_O added to 20 μL. The PCR product was extracted from 2% agarose gel and purified using the AxyPrep DNA Gel Extraction Kit (Axygen Biosciences, Union City, CA, United States), according to the manufacturer’s instructions and quantified using Quantus™ Fluorometer (Promega, Madison, WI, United States).

After amplification, purified amplicons were pooled equimolarly and paired-end sequenced (2 × 300 bp) on an Illumina MiSeq PE300 platform (Illumina, San Diego, CA, United States) by Majorbio Bio-Pharm Technology Co. Ltd. (Shanghai, China).

The raw 16S rDNA gene sequencing reads were demultiplexed and quality-filtered by Trimmomatic and merged by FLASH (version 1.2.7) according to the following criteria: (1) the 300-bp reads were truncated at any site receiving an average quality score of <20 over a 50 bp sliding window, and reads shorter than 50-bp or containing ambiguous characters were discarded; (2) only overlapping sequences longer than 10-bp were assembled according to their overlapped sequence. The maximum mismatch ratio of overlap region was 0.2. Reads that could not be assembled were discarded; and (3) individual samples were distinguished according to the barcode (exactly matching) and primers (allowing 2 nucleotide mismatches) in primer matching.

### Statistical analyses

Data were analyzed according to a 2 × 4 factorial arrangement in two concurrent 4 × 4 Latin squares using the mixed model procedure of SAS statistical package (SAS version 9.4, SAS Inst. Inc., Cary, NC). The design was not orthogonal, as there were 4 rows, 6 columns and 4 treatments, with one treatment measured three times per period (row). However, the effect of period was not significant, and, therefore, could be omitted (Sarraseca et al., 1998). Dietary energy level, animal species, and the interaction were fixed effects, and experimental animal was a random effect. When there was a significant interaction between energy level and animal species, a *t*-test was used to compare the measured variable between animal species at the same energy level. Polynomial contrasts were used to determine whether the effect of energy level on the measured variable was linear or quadratic. A level of *P* < 0.05 was accepted as significant and a trend at 0.05 ≤ *P* < 0.10.

The α-diversity (observed operational taxonomic units – OTU) for the rumen bacterial community of different energy levels were calculated with QIIME (Version 1.9.1) and analyzed by the Kruskal-Wallis test and Wilcoxon rank test using R package. The constrained principal coordinate analysis (CPCoA) was used to visualize classical multi-dimensional scaling of Bray-Curtis distance matrices using functions capscale and anova.cca of vegan package in R (version 3.4.1, United States), and the *P* value were calculated by permutation tests. The top 50 relatively abundant bacteria at the genus level were visualized as a heatmap using the R package “pheatmap”. Spearmen’s rank correlation tested the relationships between the relative abundances of the top 50 abundant ruminal bacteria (at genus level) and fermentation parameters (VFA concentration and enzyme activities) using the “corrplot” package in R (version 3.4.1, United States). Linear discriminant analysis effect size (LEfSe) determined the difference in rumen bacteria between species and among energy levels by coupling the Kruskal-Wallis Test for statistical significance with additional tests accessing biological consistency and effect relevance. Taxa with an LDA Score > 3 were considered as exhibiting a significant effect size. PICRUSt2 software predicted microbiota function and determined the differences between species and among energy levels.

## Results

### Ruminal pH, fermentation parameters and enzyme activities

Ruminal pH (average, 6.96 vs. 6.81, *P* = 0.036), ammonia-N concentration (average, 5.61 vs. 5.08 mg/dL, *P* = 0.041), and total VFAs concentration (average, 78.6 vs. 74.4 mM, *P* < 0.001) were greater in yaks than cattle (Table 2). The molar proportion of acetate (average, 73.4 vs. 72.5 mol/100 mol, *P* = 0.049) was greater, whereas of propionate was lesser (average, 15.2 vs. 15.6 mol/100 mol, *P* = 0.043), and, as a result, the ratio of acetate to proportionate (A:P) was greater (average, 4.85 vs. 4.70, *P* = 0.048) in yaks than cattle. There was no difference (*P* > 0.10) in molar proportions of butyrate, isobutyrate, valerate, and isovalerate between yaks and cattle. With increasing energy level of the diet, ruminal pH decreased linearly (*P* < 0.001), whereas the concentrations of ruminal ammonia-N and total VFAs increased linearly (*P* < 0.001) in both bovine species. The molar proportion of acetate and the ratio of A:P decreased linearly (*P* < 0.001), while the molar proportions of propionate, butyrate, isobutyrate, and isovalerate increased linearly (*P* < 0.05) with increasing energy level. The molar proportion of valerate tended to increase linearly with increasing energy level (*P* = 0.07). There was no interaction between bovine species and dietary energy level for ruminal pH and fermentation parameters (*P* > 0.10). In addition, the activities of ruminal CMCase, amylase, xylanase, and pectinase were not affected by bovine species, energy level, or their interactions (*P* > 0.10).

**TABLE 2:** Ruminal fermentation parameters and enzyme activities in yaks and cattle offered diets of different energy levels.

Items	Species	Dietary ME level, MJ/kg DM^1^				SEM	*P-value^2^*				
				
		6.62	8.02	9.42	10.80		S	E	S × E	E-L	E-Q
pH	Yak	7.20	7.02	6.84	6.76	0.090	0.036	< 0.001	0.897	< 0.001	0.412
	Cattle	7.02	6.86	6.70	6.64						
Ammonia-N mg/100mL	Yak	3.93	4.89	6.46	7.14	0.200	0.041	< 0.01	0.331	< 0.01	0.210
	Cattle	3.43	4.47	6.10	6.33						
Total VFA, mM	Yak	70.0	77.1	81.9	85.5	1.04	< 0.001	<0.001	0.330	< 0.01	<0.001
	Cattle	67.0	73.4	76.9	80.2						
**VFA, mol/100mol**											
Acetate (A)	Yak	76.1	74.1	72.7	70.8	0.53	0.049	< 0.001	0.686	< 0.001	0.245
	Cattle	74.8	73.5	71.8	70.2						
Propionate (P)	Yak	13.9	15.0	15.7	16.4	0.13	0.043	< 0.001	0.673	< 0.001	0.451
	Cattle	14.6	15.1	15.8	17.0						
Butyrate	Yak	8.80	9.60	9.90	11.2	0.201	0.110	< 0.001	0.264	< 0.001	0.147
	Cattle	9.10	9.80	10.7	11.1						
Iso-butyrate	Yak	0.37	0.46	0.51	0.51	0.052	0.092	0.013	0.586	< 0.01	0.250
	Cattle	0.50	0.50	0.57	0.60						
Valerate	Yak	0.41	0.44	0.55	0.56	0.049	0.904	0.167	0.261	0.070	0.302
	Cattle	0.48	0.50	0.47	0.51						
Iso-valerate	Yak	0.41	0.45	0.60	0.61	0.067	0.148	0.018	0.791	< 0.01	0.881
	Cattle	0.53	0.54	0.66	0.68						
A:P	Yak	5.51	4.94	4.61	4.33	0.083	0.048	< 0.001	0.568	< 0.001	0.131
	Cattle	5.14	4.88	4.57	4.21						
**Digestive enzymes activities μmol mL^–1^ min^–1^**											
CMCase	Yak	0.33	0.35	0.35	0.32	0.027	0.586	0.271	0.742	0.398	0.150
	Cattle	0.32	0.32	0.35	0.30						
Amylase	Yak	9.50	13.4	13.4	8.70	0.980	0.938	0.283	0.512	0.567	0.167
	Cattle	10.7	11.5	12.4	10.4						
Xylanase	Yak	1.75	1.73	1.64	1.51	0.160	0.369	0.912	0.581	0.235	0.894
	Cattle	1.62	1.52	1.51	1.48						
Pectinase	Yak	1.97	1.98	1.94	2.12	0.150	0.752	0.891	0.492	0.523	0.647
	Cattle	1.92	1.9	1.93	2.05						

SEM, standard error of the means; ME, metabolizable energy; VFA, volatile fatty acids.

^1^n, 6 for each group.

^2^S, animal species; E, dietary energy level; E-L, Linear effect of dietary energy levels; E-Q, Quadratic effect of dietary energy levels.

### Collective sequencing data summary

A total of 1,292,848,899 raw reads were generated from the rumen fluid samples, and 3,094,821 high quality sequences remained after quality-filtering and removal of chimeric sequences. A total of 2,744 OTUs were obtained based on 97% nucleotide sequence identity analysis among reads.

**FIGURE 1:**
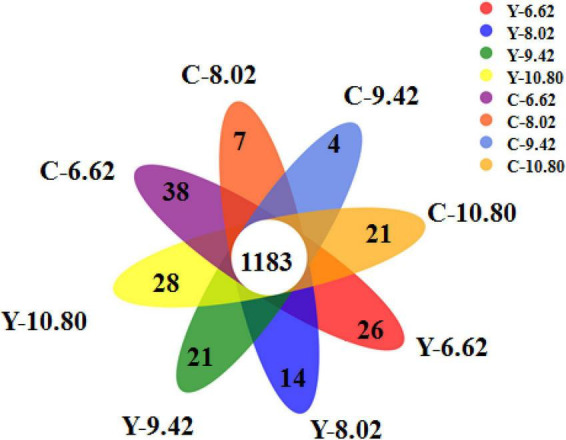
Flower plot showing different and similar OTUs in yak (Y) and Qaidam cattle (C) offered low protein diets differing energy levels (MJ ME/kg DM).

A total of 1,183 OTUs were shared among the 8 treatment groups, accounting for 56.6, 56.9, 60.1, and 59.7% of the total OTUs in yaks, and 54.6, 57.2, 58.5, and 61.4% of the total OTUs in cattle for the 6.62, 8.02, 9.42, and 10.80 MJ ME/kg DM diets, respectively (Figure 1). The number of OTUs specific to the diets containing 6.62, 8.02, 9.42, and 10.80 MJ ME/kg DM were 26, 14, 21, and 28 for yaks and 38, 7, 4, and 21 for cattle, respectively.

**FIGURE 2:**
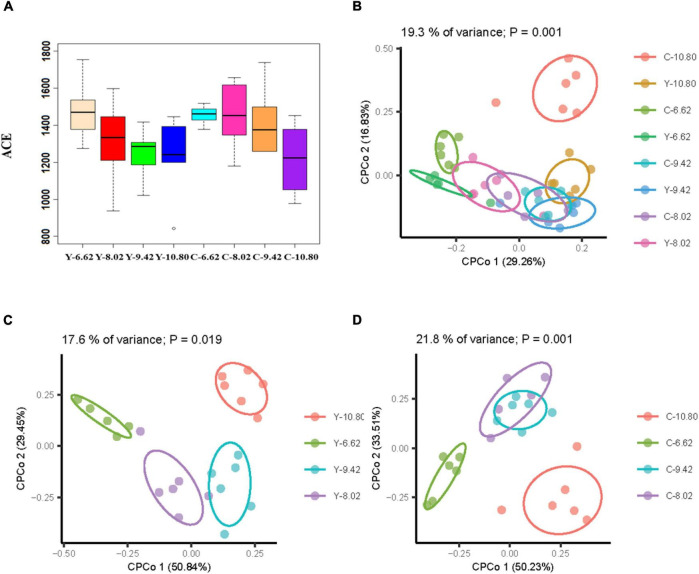
Effects of dietary energy levels on α and β diversity of the ruminal bacterial community in yak (Y) and in Qaidam cattle (C) offered low protein diets differing in energy levels (MJ ME/kg DM). **(A)** Ace index between the bovine species and among dietary energy levels; **(B)** CPCoA between the bovine species and among dietary energy levels; **(C)** CPCoA among dietary energy levels for yaks; **(D)** CPCoA among dietary energy levels for Qaidam cattle.

There was no difference in alpha diversity (ACE) between yaks and cattle, and it decreased linearly (*P* < 0.05) with increasing dietary energy level (Figure 2A). The CPCoA revealed differences in rumen bacterial communities between the bovine species and among dietary energy levels. Of the total variance, 19.3% was explained by species and dietary energy level (*P* = 0.001; Figure 2B), and 17.6% and 21.8% were explained by dietary energy level in yaks (*P* = 0.019; Figure 2C) and cattle (*P* = 0.001; Figure 2D), respectively. The rumen bacterial profiles were distinguished and affected significantly by dietary energy level, but not by bovine species (ANOSIM *R* = 0.0318, *P* = 0.098).

### Microbial community composition in the rumen fluid

**FIGURE 3:**
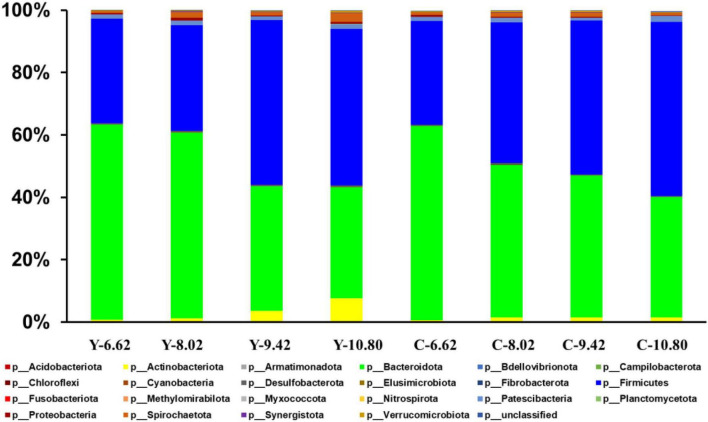
Effects of dietary energy level (MJ ME/kg DM) on the yak (Y) and Qaidam cattle (C) rumen bacterial composition at the phylum level. Each bar and color represents the average relative abundance of each phylum.

**FIGURE 4:**
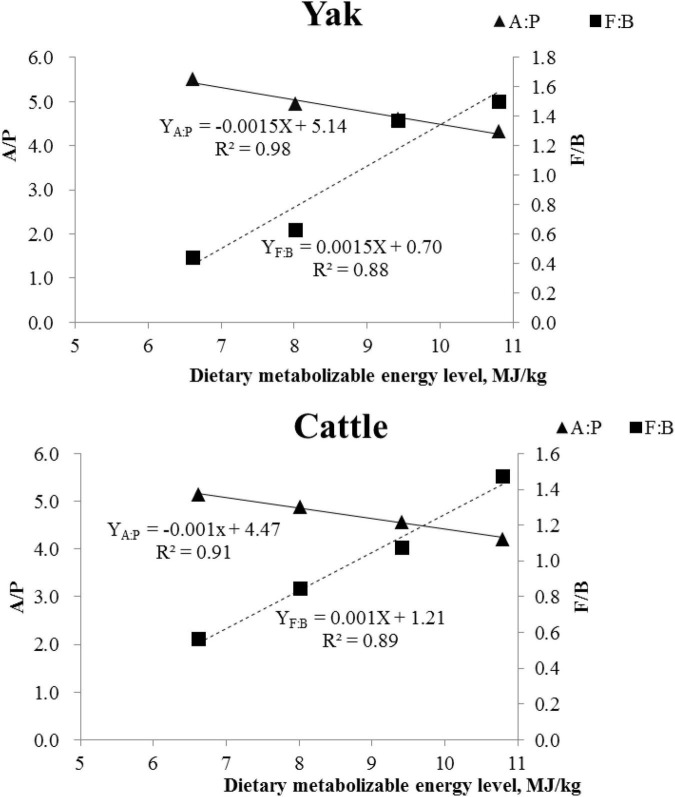
Correlation between average daily gain and Firmicutes/Bacteroidetes (F/B; ■) ratio, and acetate/propionate (A/P; ▲) ratio in yaks and in Qaidam cattle.

A total of 22 ruminal bacterial phyla were identified across the 4 energy levels and 2 animal species. The dominant phylum was Bacteroidetes with 66.9, 57.4, 38.9, and 34.7% for yaks, and 60.9, 51.0, 45.7, and 38.0% for cattle for the diets containing 6.62, 8.02, 9.42, and 10.80 MJ ME/kg DM, respectively (Figure 3 and Supplementary Table 1). Firmicutes was the second dominant phylum with 29.1, 35.9, 53.3, and 50.9% in yaks and 34.3, 43.1, 49.1, and 56.2% in cattle for the diets containing 6.62, 8.02, 9.42, and 10.80 MJ ME/kg DM, respectively. The RAs of Firmicutes and Actinobateriota increased linearly (*P* < 0.05), whereas the RA of Bacteroidetes decreased linearly (*P* < 0.05) with increasing dietary energy level. Dietary energy level was correlated negatively with the A:P ratio and positively with the Firmicutes to Bacteroidetes (F:B) ratio in both yaks and cattle (Figure 4).

**FIGURE 5:**
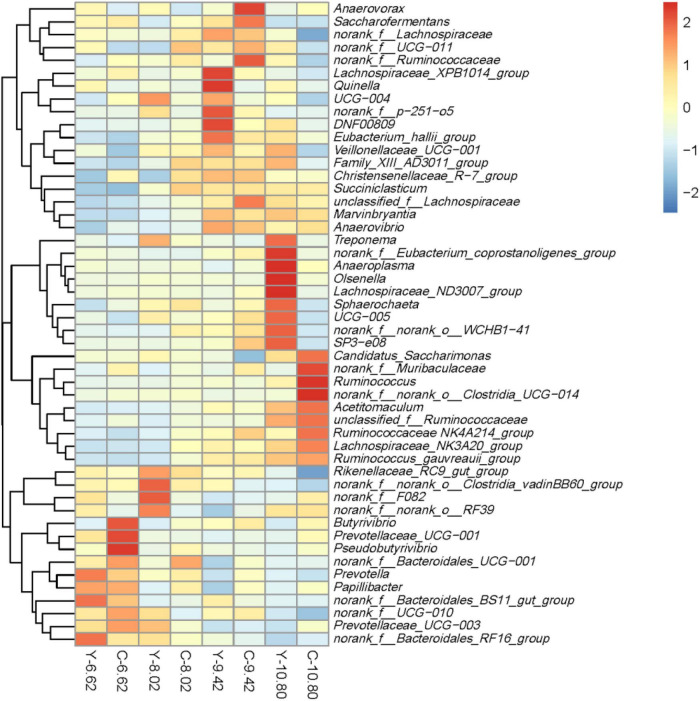
Relative abundances of the top 50 bacterial sequence variants at the genus level in the rumen of yaks (Y) and Qaidam cattle (C) fed diets differing in energy level (MJ ME/kg DM).

In total, 356 bacterial genera were identified. The dominant genera were *Prevotella* with 17.3% for yaks and 18.8% for cattle, and *Rikenellaceae_RC9_gut_group* with 16.3% for yaks and 14.9% for cattle (Figure 5 and Supplementary Table 2). The RAs of *Ruminococcaceae NK4A214_group*, *Butyrivibrio*, and *norank_f__Muribaculaceae* were lesser (*P* < 0.05), whereas, the RAs of *Rikenellaceae_RC9_gut_group* and *Quinella* were greater (*P* < 0.05) in yaks than in cattle. Moreover, the RAs of *Ruminococcaceae NK4A214_group*, *Succiniclasticum*, *Ruminococcus*, *Lachnospiraceae_NK3A20_group*, *Acetitomac ulum*, *DNF00809*, *unclassified_f__Lachnospiraceae*, *Marvinbry antia*, *Ruminococcus gauvreauii_group*, *Anaerovibrio*, and *SP3-e08* increased linearly (*P* < 0.05), while the RAs of *Prevotella*, *Rikenellaceae_RC9_gut_group*, *Prevotellaceae_UCG-003*, *norank_f__Bacteroidales_RF16_group*, *norank_f__Bacteroi dales_BS11_gut_group*, *Prevotellaceae_UCG_001*, *norank_ f__Bacteroidales_UCG-001*, and *Papillibacter* decreased linearly (*P* < 0.05) with increasing dietary energy level.

**FIGURE 6:**
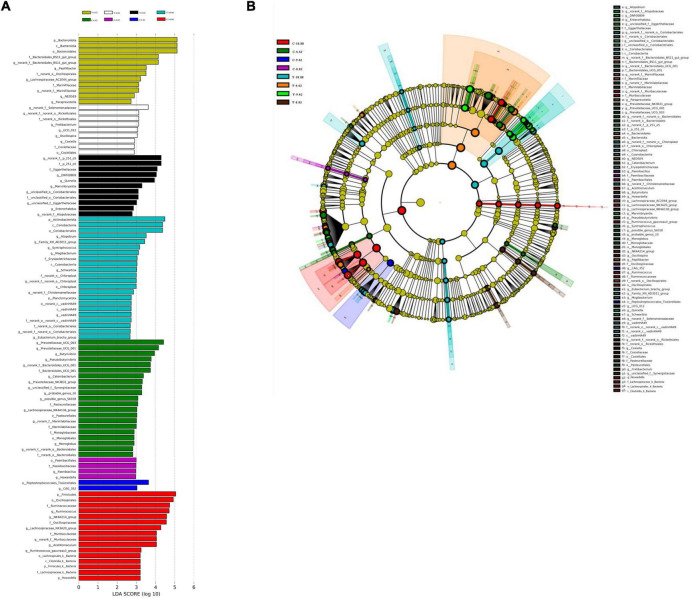
Linear discriminant analysis effect size (LEfSe) results for rumen microbiota in yaks consuming diets with different energy levels (MJ ME/kg DM). **(A)** Linear discriminant analysis. **(B)** Cladogram reported. Prefixes represent abbreviations for the taxonomic rank of each taxon, class (c^–^) order (o^–^), family (f^–^) and genus (g^–^).

Differential microbiota that varied with energy level were further identified using linear discriminant analysis effect size (LEfSe; Figures 6A,B). With a default LDA cut-off of ±2.0, differential taxa totaling 12, 9, 11, and 22 in yaks and 21, 4, 2, and 16 in cattle were observed for the 6.62, 8.02, 9.42, and 10.80 MJ ME/kg DM, respectively. The bacteria biomarkers in the 6.62 MJ ME/kg DM group were Bacteroidota for yaks and *Prevotellaceae_UCG-003*, *Prevotellaceae_UCG-001*, *Butyrivibrio*, and *Pseudobutyrivibrio* for cattle and in the 10.80 MJ ME/kg DM group were Actinobacteriota, *Atopobium*, *Family_XIII_AD3011_group*, *Syntrophococcus*, *Mogibacterium*, *Schwartzia*, *norank_f_no rank_o_chloroplast*, *norank_f_Christensenellaceae, vadinHA49*, *norank_o_norank_o_Coriobacteriales*, and *Enbacterium_ brachy_group* for yaks and Firmicutes, *Ruminococcus*, *Lachnospiraceae_NK3A20_group*, *Acetitomaculum*, and *Ruminococcus_gauvreauii_group* for cattle.

### Correlations between ruminal bacteria and fermentation parameters

**FIGURE 7:**
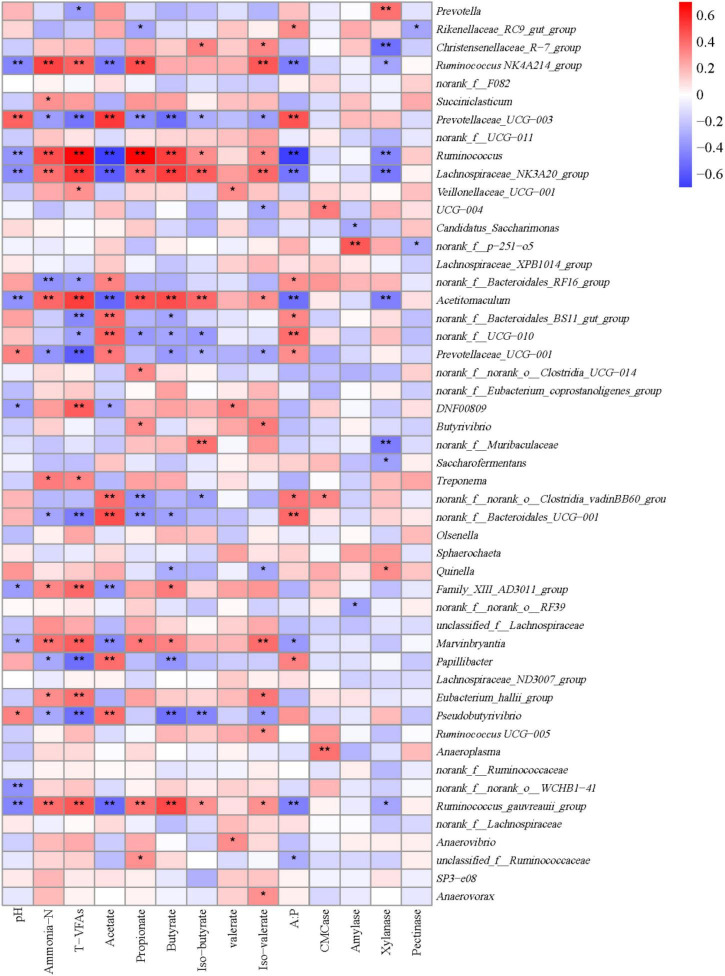
Spearman’s rank correlation analysis between the top 50 bacteria at the genus level and rumen fermentation parameters. *P < 0.05, and **P < 0.01 according to Spearman’s rank correlation coefficient.

A Spearman rank correlation tested the relationships between ruminal microbiota and rumen fermentation parameters (Figure 7). A total of 83 positive (*P* < 0.05) and 74 negative (*P* < 0.05) correlations emerged (Figure 7). *Prevotella* was correlated positively with xylanase activities (*r* = 0.385; *P* < 0.001) and negatively with the concentration of total VFAs (*r* = –0.353; *P* = 0.016). *Rinenellaceae_RC9_gut group* was correlated positively with the A:P ratio (*r* = 0.311; *P* = 0.035), but negatively with the molar proportion of propionate (*r* = –0.331; *P* = 0.025) and pectinase activities (*r* = –0.328; *P* = 0.025). *Christensenellaceae_R-7_group* was correlated positively with the molar proportions of iso-butyrate (*r* = 0.335; *P* = 0.023) and iso-valerate (*r* = 0.329; *P* = 0.026), and negatively with xylanase activities (*r* = –0.505; *P* < 0.001). *Ruminococcaceae NK4A214_group* was correlated positively with the ruminal concentrations of ammonia-N (*r* = 0.511; *P* < 0.001) and total VFAs (*r* = 0.431; *P* = 0.003) and molar proportions of propionate (*r* = 0.479; *P* = 0.001) and isovalerate (*r* = 0.447; *P* = 0.002), but negatively with ruminal pH (*r* = –0.439; *P* = 0.002), the molar proportion of acetate (*r* = –0.485; *P* = 0.001), the ratio of A:P (*r* = –0.473; *P* = 0.001), and xylanase activities (*r* = –0.327; *P* = 0.026). *Prevotellaceae_UCG-003* was correlated positively with ruminal pH (*r* = 0.425; *P* = 0.003), the molar proportion of acetate (*r* = 0.542; *P* < 0.001), and the A:P ratio (*r* = 0.460; *P* = 0.001), and negatively with the ruminal concentrations of ammonia-N (*r* = –0.358; *P* = 0.018) and total VFAs (*r* = –0.498; *P* < 0.001), and the molar proportions of propionate (*r* = –0.392; *P* = 0.007), butyrate (*r* = –0.510; *P* < 0.001), isobutyrate (*r* = –0.296; *P* = 0.046), and isovalerate (*r* = –0.336; *P* = 0.023). *Ruminococcus*, *Lachnospiraceae_NK3A20_group*, *Acetitomaculum*, and *Ruminococcus_gauvreauii_group* were correlated positively with the ruminal concentrations of ammonia-N and total VFAs, and the molar proportions of propionate, butyrate, isobutyrate, and isovalerate, but negatively with pH, the molar proportion of acetate, the A:P ratio, and xylanase activities. *Prevotellaceae_UCG-001* was correlated positively with ruminal pH (*r* = 0.324; *P* = 0.020), the molar proportion of acetate (*r* = 0.362; *P* = 0.014) and the ratio of A:P (*r* = 0.315; *P* = 0.033), but negatively with the ruminal concentrations of ammonia-N (*r* = –0. 380; *P* = 0.011) and total VFAs (*r* = –0.578; *P* < 0.001), and molar proportions of butyrate (*r* = –0.374; *P* = 0.010), isobutyrate (*r* = –0.296; *P* = 0.046), and isovalerate (*r* = –0.331; *P* = 0.025). *DNF00809* was correlated positively with the concentration of total VFAs (*r* = 0.436; *P* = 0.002) and the molar proportion of valerate (*r* = 0.335; *P* = 0.023), and negatively with ruminal pH (*r* = –0.334; *P* = 0.023) and the molar proportion of acetate (*r* = –0.322; *P* = 0.030). *Butyrivibrio* was correlated positively with the molar proportions of propionate (*r* = 0.352; *P* = 0.016) and isovalerate (*r* = 0.311; *P* = 0.035).

### PICRUSt2 function prediction

**FIGURE 8:**
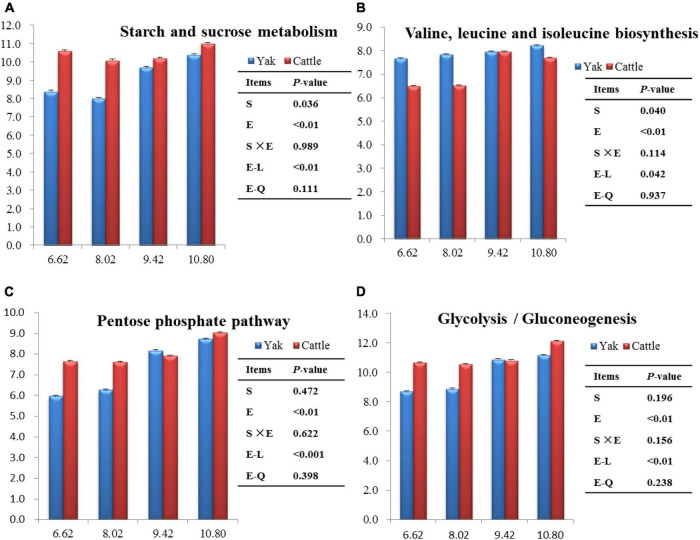
Significant differences of the functional pathways between species and among dietary energy levels (MJ ME/kg DM).

The top 40 functions of the rumen bacterial communities in yaks and cattle were predicted. There were 19 and 3 predictive metabolic pathways that were affected (*P* < 0.05) by energy level and species, respectively, and there was no interaction between species and energy level (*P* > 0.10; Supplementary Table 3). Overall, the most abundant pathway was ribosome (26.0%), followed by ABC transporters (24.8%), and then purine metabolism (22.2%). Starch and sucrose metabolisms were lesser (*P* < 0.05), whereas valine, leucine and isoleucine biosyntheses were greater (*P* < 0.05) in yaks than in cattle (Figures 8A,B). Starch and sucrose metabolisms, valine, leucine and isoleucine biosyntheses, glycolysis/gluconeogenesis, and the pentose phosphate pathway increased linearly (*P* < 0.01) with increasing dietary energy level (Figures 8C,D).

## Discussion

### Effect of dietary energy on rumen fermentation parameters

Ruminal pH, which is influenced by the concentrations of VFAs and ammonia-N and by saliva secretions, affect the growth and proliferation of ruminal microbes. In the current study, the greater ruminal pH in yaks than in cattle across the 4 energy levels was inconsistent with the greater concentration of VFAs in yaks. It was reported that the chewing activity was greater in yaks than cattle (Ding et al., 2008; Thanner et al., 2014), and this could have led to a greater saliva secretion to the rumen in yaks than in cattle. In addition, the endogenous urea recycled to the rumen was reported to be greater in yaks than in cattle (Zhou et al., 2017). The urea is hydrolyzed to ammonia, which increases the ruminal pH. Furthermore, yaks have 36 up-regulated genes related to VFA transport and absorption compared with cattle (Zhang et al., 2016), which could increase the rate of VFA absorption and reduce the ruminal concentration of VFAs. The linear decrease in ruminal pH with an increase in energy level is consistent with the linear increase in concentration of total VFAs (Liu et al., 2021b).

Ruminal ammonia-N concentration was greater in yaks than in cattle, which implied a greater microbial protein synthesis in yaks than in cattle (Zhou et al., 2017). However, the concentrations in both bovine species consuming the 6.62 and 8.02 MJ ME/kg DM diets were below the optimal range of 5 to 25 mg/100 mL (Preston and Leng, 1987), which suggests reduced activities of fibrolytic bacteria. The ammonia-N concentration increased linearly with increasing energy level, which indicated increased protein degradability and more N available for rumen microbial production, although the N intake was similar for all energy levels (Zhou et al., 2019).

Rumen VFAs are the end products of microbial fermentation, and are the main energy source for the host animal. The greater concentration of total VFAs in yaks than in cattle is in agreement with previous findings in an *in vivo* study when the two species were co-grazing a *Kobresia* pasture (Huang et al., 2012), and in an *in vitro* study when the same substrate was used with rumen inocula (Zhang et al., 2016). Furthermore, an ultra-deep metagenomic sequencing study demonstrated greater VFA-yielding pathways of rumen microbial genes in yaks than in cattle (Zhang et al., 2016). These findings could explain, at least in part, the greater ruminal concentration of VFAs in yaks than in cattle. The increase in concentration of ruminal total VFAs with increasing energy level was expected, as the non-fiber carbohydrate (NFC) contents of the concentrate increased (Liu et al., 2021b). A high-forage diet increased the molar proportion of acetate, whereas, a high concentrate diet increased the molar proportions of propionate and butyrate (Wang et al., 2019a). In the present study, the molar proportion of acetate decreased, whereas, of propionate and butyrate increased when the dietary energy increased. The greater molar proportion of acetate, along with the greater A:P ratio in yaks than in cattle, indicated a greater fiber digestibility in yaks (Wang, 2009), which was supported by the greater RA of fibrolytic bacteria (for example, *Rikenellaceae_RC9_gut_group*) in yaks than in cattle. Isobutyrate and isovalerate are derived mainly from hydrolyzation of branched-chain amino acids (BCAA; Apajalahti et al., 2019) and they are particularly important for fibrolytic bacteria (Van Gylswyk, 1970). The linear increase in iso-VFAs with increasing energy level indicated an increased protein degradability (Liu et al., 2022).

### Effect of dietary energy on bacterial community composition

Bacteroidetes and Firmicutes were the dominant bacteria phyla, as was reported in cattle (Xin et al., 2019), dairy cows (Wang et al., 2019b), goats (Xue et al., 2022), and sheep (Castro-Carrera et al., 2014). These findings suggest that these two phyla play important roles in feed digestion and metabolism in the rumen. The mean RAs of Firmicutes for yaks (42.3%) and cattle (45.7%), and of Bacteroidetes for yaks (49.5%) and cattle (48.9%) were either below (56% for Firmicutes) or above (31% for Bacterioidetes) values from a meta-analysis of all curated 16S rRNA gene sequences from a NCBI database for ruminants (Kim et al., 2011). This could be due to differences in animal species, growth stage, and dietary nutritional levels and composition among studies. In the present study, the RA of Bacteroidetes decreased linearly and of Firmicutes increased linearly with increasing energy level, as was also reported in other yaks (Hu et al., 2020) and in goats (Min et al., 2019). The increased F:B ratio with increasing energy levels is in agreement with Liu et al. (2022), who also reported a linear increase in average daily gain (ADG) in yaks and cattle with increasing energy levels. A lower ratio of F:B was linked to an inhibition of fat deposition and decreased ADG of the host animal (Liu et al., 2015; Hu et al., 2020).

The maximum RA of Actinobacteria, a gram-positive bacteria, was 3% for both cattle and sheep (Sul’ák et al., 2012), and decreased with increasing energy level in Holstein heifers (Bi et al., 2018). In the present study, there was a larger variation in the RA of Actinobacteria (0.79% to 7.63%) in yaks than in cattle (0.66% to 1.57%), which suggests that Actinobacteria may be more sensitive to dietary energy change in yaks than in cattle.

*Prevotella* was the most dominant genus, which is in agreement with previous studies in steers (Xin et al., 2019), dairy cows (Wei et al., 2021), goats (Dao et al., 2021), and sheep (Huang et al., 2022). In the present study, *Prevotella* contained a number of metabolic versatile species, which secrete enzymes to degrade protein, starch, peptides, hemicellulose, and pectin into VFAs and amino acids (Stevenson and Weimer, 2007; Dao et al., 2021). This variability can be associated with the maintenance of RA balance under different fermentation conditions. The RA of *Prevotella* decreased linearly with increasing energy level, which is in agreement with a study in Angus cows (Chen et al., 2021). In addition, the RAs of *Prevotellaceae_UCG-001* and *Prevotellaceae_UCG-003*, which belong to the Prevotellaceae family, also decreased linearly with increasing energy level. The reduction in RAs of these bacteria could be explained by a decrease in dietary fiber contents with increasing energy level. The *Rikenellaceae_RC9_gut_group* was the second most dominant genus, which is consistent with a study in grazing yaks (Liu et al., 2021a) and cattle (Xin et al., 2019). *Rikenellaceae_RC9_gut_group*, a member of the Rikenellaceae family, degrades cellulose and hemicellulose, with acetate as the main end product (Sha et al., 2020), and this could explain the linear decrease in RA with an increase in energy level (i.e., decrease in fiber content). The greater RA of *Rikenellaceae_RC9_gut_group* in yaks than in cattle could explain, at least in part, the greater fiber digestibility (Liu et al., 2022) and greater molar proportion of acetate in yaks than in cattle.

*Ruminococcus* is a fibrolytic bacteria and occurs predominantly in the rumen of ruminants fed high-fiber diets (Wei et al., 2021). However, in the present study, the RA of *Ruminococcus* increased linearly with increasing energy level and decreasing fiber content. This genus also exhibits amylolytic activity and the increasing dietary energy could have stimulated its grow and proliferation (Hu et al., 2020). This could explain why *Ruminococcus* was associated positively with total VFAs concentration, and negatively with pH. *Ruminococcus_gauvreauii_group*, which increased with increasing energy level in the present study, is a gram-positive and obligate anaerobic bacteria that produces acetate as the main end-product of glucose fermentation (Domingo et al., 2008). It was correlated negatively with pH and acetate and positively with the molar proportion of propionate. The ammonia-N concentrations in both bovine species consuming the 6.62 and 8.02 MJ ME/kg DM diets were below the optimal range of 5 mg/100 mL, which could explain why the proliferation of *Ruminococcus_gauvreauii_group* was inhibited. *Ruminococcus_NK4A214 group*, another bacterium belonging to the Ruminococcus family, was associated positively with total VFAs, and the molar proportion of propionate, which is in agreement with Li et al. (2019).

*Succiniclasticum* utilizes succinate to produce propionate (Van Gylswyk, 1995). In the present study, the increased RA of *Succiniclasticum* with increasing energy level was likely associated with the increased dietary NFC. *Lachnospiraceae_NK3A20_group* was characterized by cellulose-decomposing activity and starch hydrolysis, which could produce acetate and formate (Russell and Rychlik, 2001). In addition, *Lachnospiraceae_NK3A20_group* was correlated positively with total VFAs concentration, and the molar proportions of propionate, butyrate, isobutyrate, and isovalerate, and negatively with pH, the molar proportion of acetate, the ratio of A:P, and xylanase activities. These results suggest that the fermentation pattern of *Lachnospiraceae_NK3A20_group* favor propionate production in high-energy diets when consuming a low protein diet. A low concentration of ammonia-N could inhibit the proliferation of this genus, which could explain this occurrence. *Acetitomaculum* utilizes monosaccharides to produce acetate and occurs mainly in ruminants fed a high-concentrate diet (Huang C. et al., 2021). Accordingly, the RA of *Acetitomaculum* increased with increasing energy level, which was in line with Wang et al. (2019a).

*Quinella* ferments sugars equimolarly to acetate and propionate. It was reported that a greater RA of *Quinella* was associated with a lower methane emission in ruminants (Krumholz et al., 1993). The greater RA of *Quinella* in yaks than in cattle could explain, at least in part, the lesser methane emissions from yaks than cattle (Mi et al., 2017; Bai et al., 2021). In agreement with a report by Mao et al. (2016), the RA of *Papillibacter* decreased with increasing energy levels. *Papillibacter* was correlated positively with the molar proportion of acetate and the ratio of A:P, and negatively with total VFAs concentration and the molar proportion of butyrate.

To further understand the effects of energy level and bovine species on the bacterial community, we used LEfSe analysis. Bacteroidetes was the most differentially abundant bacteria in the yaks fed the low energy (6.62 MJ ME/kg DM) diet, inferring that Bacteroidetes was important for yaks with sparse forage availability. In addition, the RA of Firmicutes was greatest in cattle consuming the high energy (10.40 MJ ME/kg DM) diet, suggesting that a high-quality diet was linked with this phylum (Hu et al., 2020).

### Correlations between bacterial communities at genera level and fermentation parameters and PICRUSt2 prediction of functions

The *Rikenellaceae_RC9_gut_group* was correlated negatively with the molar proportion of propionate and pectinase activity, and positively with the A:P ratio, suggesting fibrolytic activity, which is in agreement with previous studies (Zhao et al., 2017; Wang et al., 2019a). In the present study, *Pseudobutyrivibrio* was correlated positively with the molar proportion of acetate and negatively with total VFAs concentration, which is in accordance with the reports that this bacterium is cellulotytic (Poonko et al., 2015). Therefore, it was correlated positively with dietary fiber content and negatively with dietary protein and starch (Hao et al., 2021). The reason for the negative correlation of *Prevotella*, *Prevotellaceae_UCG-001*, and *Prevotellaceae_UCG-003* with total VFAs concentration is not clear. *Prevotella* degrades most fibers and protein, but its function and metabolism are still unknown. There were 11 ruminal bacteria that were not associated with rumen fermentation parameters.

By using PICRUSt2 to predict the potential functions of bacteria, numerous metabolism related pathways emerged. The most prominent functional categories were ribosome and ABC transporters at the KEGG level 3 metabolic categories. These functions are vital for the survival, reproduction, and growth of livestock (Lamendella et al., 2011). In the present study, there were 17 predictive metabolic pathways that were affected by energy levels. The BCAAs, including valine, leucine, and isoleucine, are vital contributors to microbial protein synthesis (Allison et al., 1966). Ruminal microbial protein supplies up to 85% of the total amino acids absorbed in the small intestine (Westreicher-Kristen et al., 2020). In the present study, molar proportions of valine, leucine and isoleucine were greater in yaks than in cattle, and increased linearly with increasing energy level, inferring that BCAA biosynthesis in the rumen of yaks produce more microbial protein than in cattle (Zhou et al., 2018). The *Prevotella* genus has a strong relationship with BCAA synthesis (Xue et al., 2020). However, in the present study, no difference in the RA of *Prevotella* was observed between yaks and cattle.

## Conclusion

The greater ruminal concentration of total VFAs and acetate to propionate ratio in yaks than in cattle was supported by the greater RA of fibrolytic bacteria, such as *Rikenellaceae_RC9_gut_group*. In addition, Bacteroidetes and Firmicutes were the dominant phyla, and *Prevotella* and *Rikenellaceae_RC9_gut_group* were the dominant genera, regardless of energy level and bovine species. The different bacterial profiles between yaks and Qaidam cattle explain how yaks are better adapted to the poor forage on the QTP than cattle. This study provides insight in the response of ruminal bacteria in yaks and cattle to a low protein diet with differing energy levels, as is common at high altitudes.

## Data availability statement

The datasets presented in this study can be found in online repositories. The names of the repository/repositories and accession number(s) can be found below: NCBI SRA BioProject, accession no: PRJNA856780.

## Ethics statement

The protocol and procedures on the animals were approved by the Animal Care and Use Committee of Lanzhou University, Gansu, China (No. 201905201).

## Author contributions

JZ and RL: conceptualization. HL: methodology, formal analysis, data curation, and writing – original draft preparation. ZS and TR: software. JZ: validation and project administration. HL and TR: investigation. CZ: resources. JZ, ZS, TR, WY, and AD: writing – review and editing. ZS and HL: visualization. TR: supervision. JZ, TR, and RL: funding acquisition. All authors reviewed the manuscript.
