# Supplementary material for: Comparison of rumen bacterial communities between yaks (Bos grunniens) and Qaidam cattle (Bos taurus) fed a low protein diet with different energy levels
Source: Front Microbiol. 2022 Sep 6;13:982338. doi: 10.3389/fmicb.2022.982338 (PMC9486477; doi:10.3389/fmicb.2022.982338)
Supplement: Supplementary file 1 [file Table_1.docx]

Table S1 The relative abundances (%) of bacteria at the phylum level in yaks and cattle offered diets of different energy levels.

| Items | Species | Dietary ME level, MJ/kg DM | | | | SEM | *P*-values^1^ | | | | |
| --- | --- | --- | --- | --- | --- | --- | --- | --- | --- | --- | --- |
|  |  | LE | MLE | MHE | HE |  | S | E | S × E | E-L | E-Q |
| Bacteroidetes | Yak | 66.9 | 57.4 | 38.9 | 34.7 | 5.71 | 0.748 | <0.01 | 0.553 | <0.001 | 0.568 |
|  | Cattle | 60.9 | 51.0 | 45.7 | 38.0 |  |  |  |  |  |  |
| Firmicutes | Yak | 29.1 | 35.9 | 53.3 | 50.9 | 5.55 | 0.346 | <0.01 | 0.685 | <0.01 | 0.487 |
|  | Cattle | 34.3 | 43.1 | 49.1 | 56.2 |  |  |  |  |  |  |
| Actinobacteriota | Yak | 0.79 | 1.21 | 3.77 | 7.63 | 1.338 | 0.063 | 0.226 | 0.089 | 0.045 | 0.916 |
|  | Cattle | 0.66 | 1.39 | 1.58 | 1.57 |  |  |  |  |  |  |
| Patescibacteria | Yak | 1.35 | 1.41 | 1.18 | 1.54 | 0.508 | 0.942 | 0.781 | 0.850 | 0.696 | 0.357 |
|  | Cattle | 1.50 | 1.38 | 0.78 | 1.99 |  |  |  |  |  |  |
| Spirochaetota | Yak | 0.74 | 1.99 | 1.07 | 2.94 | 0.553 | 0.228 | 0.115 | 0.131 | 0.157 | 0.995 |
|  | Cattle | 0.96 | 1.65 | 1.28 | 0.82 |  |  |  |  |  |  |
| Proteobacteria | Yak | 0.27 | 0.85 | 0.36 | 0.61 | 0.208 | 0.287 | 0.879 | 0.125 | 0.469 | 0.795 |
|  | Cattle | 0.55 | 0.22 | 0.39 | 0.19 |  |  |  |  |  |  |
| Verrucomicrobiota | Yak | 0.21 | 0.24 | 0.31 | 0.62 | 0.104 | 0.561 | 0.475 | 0.012 | 0.289 | 0.246 |
|  | Cattle | 0.22 | 0.38 | 0.42 | 0.15 |  |  |  |  |  |  |
| Desulfobacterota | Yak | 0.28 | 0.28 | 0.34 | 0.28 | 0.067 | 0.588 | 0.977 | 0.705 | 0.703 | 0.900 |
|  | Cattle | 0.25 | 0.32 | 0.22 | 0.27 |  |  |  |  |  |  |
| unclassified_k__  norank_d__Bacteria | Yak | 0.03 | 0.08 | 0.13 | 0.15 | 0.110 | 0.300 | 0.354 | 0.909 | 0.085 | 0.977 |
|  | Cattle | 0.03 | 0.15 | 0.23 | 0.31 |  |  |  |  |  |  |
| Synergistota | Yak | 0.09 | 0.26 | 0.12 | 0.11 | 0.065 | 0.252 | 0.352 | 0.742 | 0.962 | 0.119 |
|  | Cattle | 0.05 | 0.13 | 0.12 | 0.04 |  |  |  |  |  |  |

SEM = standard error of the means; ME = metabolizable energy.

^1^ S = species; E = dietary energy level; E-L = linear effect of dietary energy levels; E-Q = quadratic effect of dietary energy levels.

Table S2 The relative abundances (%) of bacteria at the genus level in yaks and cattle offered diets of different energy levels.

| Items | Species | Dietary ME level, MJ/kg DM | | | | SEM | *P*-values^1^ | | | | |
| --- | --- | --- | --- | --- | --- | --- | --- | --- | --- | --- | --- |
|  |  | LE | MLE | MHE | HE |  | S | E | S × E | E-L | E-Q |
| *Prevotella* | Yak | 27.20 | 17.85 | 12.29 | 13.70 | 2.46 | 0.569 | <0.001 | 0.248 | <0.001 | 0.252 |
|  | Cattle | 24.05 | 20.88 | 17.89 | 12.36 |  |  |  |  |  |  |
| *Rikenellaceae_RC9_gut_group* | Yak | 17.20 | 19.33 | 15.39 | 13.84 | 0.46 | 0.029 | 0.011 | 0.491 | 0.011 | 0.027 |
|  | Cattle | 15.32 | 17.54 | 16.09 | 12.49 |  |  |  |  |  |  |
| *Christensenellaceae_R-7_group* | Yak | 3.22 | 3.17 | 7.67 | 5.51 | 0.89 | 0.312 | 0.142 | 0.112 | 0.282 | 0.232 |
|  | Cattle | 5.92 | 6.92 | 7.50 | 5.27 |  |  |  |  |  |  |
| *Ruminococcaceae NK4A214_group* | Yak | 3.27 | 3.11 | 5.34 | 5.37 | 0.75 | 0.013 | <0.001 | 0.013 | <0.001 | 0.714 |
|  | Cattle | 2.37 | 4.67 | 7.50 | 9.68 |  |  |  |  |  |  |
| *norank_f__F082* | Yak | 6.07 | 9.73 | 2.14 | 2.98 | 0.73 | 0.244 | 0.082 | 0.077 | 0.357 | 0.913 |
|  | Cattle | 3.46 | 3.42 | 2.66 | 5.46 |  |  |  |  |  |  |
| *Succiniclasticum* | Yak | 1.66 | 3.24 | 4.20 | 4.11 | 0.75 | 0.538 | 0.015 | 0.777 | <0.01 | 0.070 |
|  | Cattle | 1.57 | 4.65 | 4.31 | 4.06 |  |  |  |  |  |  |
| *Prevotellaceae_UCG-003* | Yak | 5.16 | 6.15 | 1.28 | 1.35 | 0.34 | 0.548 | <0.001 | <0.01 | <0.001 | 0.089 |
|  | Cattle | 6.94 | 3.20 | 1.78 | 3.25 |  |  |  |  |  |  |
| *norank_f__UCG-011* | Yak | 3.55 | 2.09 | 3.73 | 3.55 | 0.43 | 0.942 | 0.705 | 0.133 | 0.915 | 0.565 |
|  | Cattle | 2.08 | 4.19 | 4.37 | 2.17 |  |  |  |  |  |  |
| *Ruminococcus* | Yak | 0.26 | 0.42 | 1.74 | 3.38 | 0.94 | 0.189 | <0.001 | <0.001 | <0.001 | <0.001 |
|  | Cattle | 0.57 | 1.34 | 1.22 | 5.75 |  |  |  |  |  |  |
| *Lachnospiraceae_NK3A20_group* | Yak | 0.75 | 0.85 | 2.81 | 3.70 | 0.40 | 0.524 | <0.001 | 0.005 | <0.001 | 0.070 |
|  | Cattle | 0.81 | 2.39 | 2.47 | 4.96 |  |  |  |  |  |  |
| *Veillonellaceae_UCG-001* | Yak | 1.36 | 1.96 | 2.77 | 2.79 | 0.22 | 0.171 | 0.392 | 0.537 | 0.373 | 0.143 |
|  | Cattle | 0.68 | 1.99 | 2.01 | 2.85 |  |  |  |  |  |  |
| *UCG-004* | Yak | 0.50 | 3.29 | 3.13 | 1.63 | 0.31 | 0.272 | 0.839 | 0.130 | 0.886 | 0.210 |
|  | Cattle | 1.61 | 1.32 | 1.17 | 1.13 |  |  |  |  |  |  |
| *Candidatus_Saccharimonas* | Yak | 1.20 | 1.34 | 1.13 | 1.52 | 0.41 | 0.864 | 0.683 | 0.877 | 0.376 | 0.423 |
|  | Cattle | 1.18 | 1.21 | 0.75 | 1.88 |  |  |  |  |  |  |
| *norank_f__p-251-o5* | Yak | 0.39 | 2.21 | 4.06 | 0.17 | 0.02 | 0.106 | 0.064 | 0.234 | 0.742 | 0.023 |
|  | Cattle | 0.38 | 0.44 | 1.51 | 0.31 |  |  |  |  |  |  |
| *Lachnospiraceae_XPB1014_group* | Yak | 0.92 | 0.72 | 2.87 | 0.72 | 0.20 | 0.472 | 0.040 | 0.253 | 0.587 | 0.116 |
|  | Cattle | 1.36 | 0.97 | 1.25 | 0.40 |  |  |  |  |  |  |
| *norank_f__Bacteroidales_RF16_group* | Yak | 2.08 | 1.61 | 1.03 | 0.59 | 0.02 | 0.563 | 0.100 | 0.849 | 0.015 | 0.929 |
|  | Cattle | 1.56 | 1.18 | 0.89 | 0.78 |  |  |  |  |  |  |
| *Acetitomaculum* | Yak | 0.31 | 0.43 | 1.16 | 1.99 | 0.08 | 0.478 | <0.001 | 0.390 | <0.001 | <0.01 |
|  | Cattle | 0.34 | 0.68 | 0.92 | 2.61 |  |  |  |  |  |  |
| *norank_f__Bacteroidales_BS11_gut_group* | Yak | 3.67 | 0.09 | 1.71 | 0.13 | 0.02 | 0.466 | 0.024 | 0.833 | 0.030 | 0.128 |
|  | Cattle | 2.63 | 0.39 | 0.56 | 0.11 |  |  |  |  |  |  |
| *norank_f__UCG-010* | Yak | 1.40 | 1.41 | 1.16 | 0.67 | 0.10 | 0.569 | 0.271 | 0.608 | 0.158 | 0.478 |
|  | Cattle | 1.68 | 0.75 | 1.27 | 0.45 |  |  |  |  |  |  |
| *Prevotellaceae_UCG-001* | Yak | 1.82 | 0.56 | 0.24 | 0.35 | 0.03 | 0.063 | <0.001 | 0.145 | <0.001 | <0.001 |
|  | Cattle | 3.34 | 0.50 | 0.53 | 1.21 |  |  |  |  |  |  |
| *norank_f__norank_o__Clostridia_UCG-014* | Yak | 0.62 | 0.79 | 0.59 | 0.75 | 0.15 | 0.187 | 0.419 | 0.270 | 0.208 | 0.354 |
|  | Cattle | 0.78 | 0.88 | 0.68 | 2.96 |  |  |  |  |  |  |
| *norank_f__Eubacterium_coprostanoligenes_group* | Yak | 0.79 | 0.59 | 0.78 | 1.83 | 0.17 | 0.310 | 0.448 | 0.214 | 0.235 | 0.300 |
|  | Cattle | 0.67 | 0.58 | 0.97 | 0.69 |  |  |  |  |  |  |
| *DNF00809* | Yak | 0.32 | 0.36 | 2.35 | 1.28 | 0.09 | 0.068 | <0.01 | 0.053 | 0.043 | 0.146 |
|  | Cattle | 0.37 | 0.58 | 0.84 | 0.36 |  |  |  |  |  |  |
| *Butyrivibrio* | Yak | 0.40 | 0.37 | 0.84 | 0.33 | 0.10 | <0.01 | 0.219 | 0.077 | 0.380 | 0.246 |
|  | Cattle | 2.07 | 0.75 | 1.12 | 0.99 |  |  |  |  |  |  |
| *norank_f__Muribaculaceae* | Yak | 0.34 | 0.24 | 0.42 | 0.42 | 0.08 | 0.023 | 0.237 | 0.408 | 0.202 | 0.114 |
|  | Cattle | 1.06 | 0.62 | 1.22 | 2.52 |  |  |  |  |  |  |
| *Saccharofermentans* | Yak | 0.90 | 0.58 | 0.95 | 0.55 | 0.07 | 0.412 | 0.019 | 0.842 | 0.257 | 0.264 |
|  | Cattle | 0.89 | 0.77 | 1.21 | 0.55 |  |  |  |  |  |  |
| *Treponema* | Yak | 0.59 | 1.25 | 0.64 | 1.46 | 0.17 | 0.214 | 0.276 | 0.531 | 0.318 | 0.995 |
|  | Cattle | 0.52 | 0.73 | 0.59 | 0.62 |  |  |  |  |  |  |
| *norank_f__norank_o__Clostridia_vadinBB60_group* | Yak | 0.81 | 1.85 | 0.83 | 0.33 | 0.02 | 0.312 | 0.311 | 0.759 | 0.267 | 0.174 |
|  | Cattle | 0.84 | 0.97 | 0.36 | 0.11 |  |  |  |  |  |  |
| *norank_f__Bacteroidales_UCG-001* | Yak | 0.78 | 0.54 | 0.19 | 0.39 | 0.10 | 0.141 | 0.071 | 0.634 | 0.014 | 0.634 |
|  | Cattle | 1.21 | 1.17 | 0.61 | 0.42 |  |  |  |  |  |  |
| *Olsenella* | Yak | 0.07 | 0.30 | 0.19 | 3.54 | 0.01 | 0.365 | 0.697 | 0.466 | 0.345 | 0.558 |
|  | Cattle | 0.09 | 0.20 | 0.10 | 0.28 |  |  |  |  |  |  |
| *Sphaerochaeta* | Yak | 0.14 | 0.72 | 0.43 | 1.47 | 0.04 | 0.750 | 0.464 | 0.298 | 0.297 | 0.995 |
|  | Cattle | 0.41 | 0.89 | 0.68 | 0.19 |  |  |  |  |  |  |
| *Quinella* | Yak | 0.792 | 0.241 | 2.617 | 0.677 | 0.00 | 0.036 | 0.978 | 0.730 | 0.773 | 0.963 |
|  | Cattle | 0.007 | 0.020 | 0.003 | 0.007 |  |  |  |  |  |  |
| *Family_XIII_AD3011_group* | Yak | 0.26 | 0.32 | 0.59 | 0.68 | 0.05 | 0.769 | 0.115 | 0.127 | 0.155 | 0.048 |
|  | Cattle | 0.19 | 0.63 | 0.58 | 0.30 |  |  |  |  |  |  |
| *norank_f__norank_o__RF39* | Yak | 0.46 | 0.57 | 0.27 | 0.47 | 0.11 | 0.403 | 0.710 | 0.488 | 0.601 | 0.423 |
|  | Cattle | 0.35 | 0.34 | 0.35 | 0.47 |  |  |  |  |  |  |
| *unclassified_f__Lachnospiraceae* | Yak | 0.34 | 0.36 | 0.44 | 0.46 | 0.08 | 0.777 | 0.133 | 0.812 | 0.049 | 0.594 |
|  | Cattle | 0.32 | 0.38 | 0.54 | 0.42 |  |  |  |  |  |  |
| *Marvinbryantia* | Yak | 0.23 | 0.26 | 0.56 | 0.55 | 0.04 | 0.796 | 0.031 | 0.940 | <0.01 | 0.950 |
|  | Cattle | 0.21 | 0.32 | 0.49 | 0.50 |  |  |  |  |  |  |
| *Papillibacter* | Yak | 0.70 | 0.23 | 0.13 | 0.24 | 0.05 | 0.177 | 0.014 | 0.550 | 0.028 | 0.011 |
|  | Cattle | 0.65 | 0.46 | 0.37 | 0.37 |  |  |  |  |  |  |
| *Lachnospiraceae_ND3007_group* | Yak | 0.19 | 0.23 | 0.16 | 1.12 | 0.04 | 0.205 | 0.410 | 0.035 | 0.205 | 0.308 |
|  | Cattle | 0.23 | 0.19 | 0.29 | 0.13 |  |  |  |  |  |  |
| *Eubacterium_hallii_group* | Yak | 0.20 | 0.27 | 0.56 | 0.40 | 0.08 | 0.365 | 0.053 | 0.772 | 0.074 | 0.075 |
|  | Cattle | 0.14 | 0.30 | 0.38 | 0.27 |  |  |  |  |  |  |
| *Pseudobutyrivibrio* | Yak | 0.30 | 0.11 | 0.16 | 0.04 | 0.02 | 0.147 | 0.342 | 0.501 | 0.121 | 0.337 |
|  | Cattle | 1.22 | 0.40 | 0.12 | 0.26 |  |  |  |  |  |  |
| *UCG-005* | Yak | 0.24 | 0.31 | 0.28 | 0.46 | 0.03 | 0.257 | 0.575 | 0.374 | 0.196 | 0.605 |
|  | Cattle | 0.18 | 0.25 | 0.34 | 0.18 |  |  |  |  |  |  |
| *Anaeroplasma* | Yak | 0.23 | 0.25 | 0.23 | 0.42 | 0.01 | 0.709 | 0.450 | 0.789 | 0.126 | 0.870 |
|  | Cattle | 0.25 | 0.26 | 0.25 | 0.27 |  |  |  |  |  |  |
| *norank_f__Ruminococcaceae* | Yak | 0.23 | 0.25 | 0.25 | 0.28 | 0.05 | 0.525 | 0.412 | 0.346 | 0.931 | 0.468 |
|  | Cattle | 0.27 | 0.30 | 0.40 | 0.19 |  |  |  |  |  |  |
| *norank_f__norank_o__WCHB1-41* | Yak | 0.15 | 0.15 | 0.27 | 0.54 | 0.01 | 0.562 | 0.440 | 0.023 | 0.175 | 0.359 |
|  | Cattle | 0.14 | 0.30 | 0.35 | 0.12 |  |  |  |  |  |  |
| *Ruminococcus_gauvreauii_group* | Yak | 0.11 | 0.10 | 0.29 | 0.38 | 0.02 | 0.517 | 0.040 | 0.921 | <0.001 | 0.870 |
|  | Cattle | 0.08 | 0.20 | 0.31 | 0.43 |  |  |  |  |  |  |
| *norank_f__Lachnospiraceae* | Yak | 0.26 | 0.22 | 0.32 | 0.23 | 0.05 | 0.574 | 0.153 | 0.739 | 0.496 | 0.175 |
|  | Cattle | 0.23 | 0.27 | 0.30 | 0.15 |  |  |  |  |  |  |
| *Anaerovibrio* | Yak | 0.11 | 0.15 | 0.34 | 0.25 | 0.09 | 0.727 | 0.064 | 0.971 | 0.035 | 0.362 |
|  | Cattle | 0.18 | 0.18 | 0.32 | 0.29 |  |  |  |  |  |  |
| *unclassified_f__Ruminococcaceae* | Yak | 0.08 | 0.06 | 0.14 | 0.44 | 0.08 | 0.683 | 0.347 | 0.943 | 0.113 | 0.412 |
|  | Cattle | 0.15 | 0.14 | 0.15 | 0.54 |  |  |  |  |  |  |
| *SP3-e08* | Yak | 0.16 | 0.16 | 0.20 | 0.44 | 0.14 | 0.464 | 0.062 | 0.071 | 0.011 | 0.595 |
|  | Cattle | 0.17 | 0.19 | 0.33 | 0.11 |  |  |  |  |  |  |
| *Anaerovorax* | Yak | 0.22 | 0.21 | 0.20 | 0.19 | 0.03 | 0.862 | 0.411 | 0.224 | 0.230 | 0.966 |
|  | Cattle | 0.17 | 0.17 | 0.31 | 0.21 |  |  |  |  |  |  |

SEM = standard error of the means; ME = metabolizable energy.

^1^ S = species; E = dietary energy level; E-L = linear effect of dietary energy levels; E-Q = quadratic effect of dietary energy levels.

Table S3. Functional predictions (the top 40 abundant KEGG at genera level) for rumen bacteria in yaks and cattle offered diets of different energy levels.

| Items | Species | Dietary ME level, MJ/kg DM | | | | SEM | *P*-value^1^ | | | | |
| --- | --- | --- | --- | --- | --- | --- | --- | --- | --- | --- | --- |
|  |  | LE | MEL | MHE | HE |  | S | E | S × E | E-L | E-Q |
| **Metabolism** | | | | | | | | | | | |
| **Carbohydrate Metabolism** | | | | | | | | | | | |
| Amino sugar and nucleotide sugar metabolism | Yak | 11.6 | 12.1 | 14.1 | 14.5 | 0.05 | 0.325 | 0.471 | 0.365 | 0.983 | 0.215 |
|  | Cattle | 14.4 | 14.2 | 14.0 | 13.8 |  |  |  |  |  |  |
| Glycolysis / Gluconeogenesis | Yak | 8.69 | 8.87 | 10.9 | 11.2 | 0.04 | 0.196 | <0.01 | 0.156 | <0.01 | 0.238 |
|  | Cattle | 10.7 | 10.5 | 10.8 | 12.1 |  |  |  |  |  |  |
| Pyruvate metabolism | Yak | 8.11 | 8.28 | 10.5 | 10.7 | 0.04 | 0.282 | <0.001 | 0.400 | <0.001 | 0.335 |
|  | Cattle | 9.82 | 10.0 | 10.4 | 11.3 |  |  |  |  |  |  |
| Starch and sucrose metabolism | Yak | 8.40 | 8.01 | 9.71 | 10.4 | 0.05 | 0.036 | <0.01 | 0.989 | <0.01 | 0.111 |
|  | Cattle | 10.6 | 10.1 | 10.2 | 11.0 |  |  |  |  |  |  |
| Fructose and mannose metabolism | Yak | 6.86 | 7.23 | 8.73 | 9.31 | 0.059 | 0.976 | 0.376 | 0.810 | 0.090 | 0.961 |
|  | Cattle | 8.69 | 8.48 | 8.57 | 9.21 |  |  |  |  |  |  |
| Pentose phosphate pathway | Yak | 5.96 | 6.27 | 8.17 | 8.73 | 0.050 | 0.472 | <0.01 | 0.622 | <0.001 | 0.398 |
|  | Cattle | 7.65 | 7.60 | 7.91 | 9.03 |  |  |  |  |  |  |
| Galactose metabolism | Yak | 6.05 | 6.16 | 7.05 | 7.22 | 0.024 | 0.819 | 0.611 | 0.517 | 0.966 | 0.611 |
|  | Cattle | 7.32 | 7.14 | 7.20 | 7.38 |  |  |  |  |  |  |
| Citrate cycle (TCA cycle) | Yak | 6.29 | 6.10 | 6.66 | 6.27 | 0.047 | 0.657 | 0.204 | 0.075 | 0.059 | 0.416 |
|  | Cattle | 6.98 | 6.77 | 6.67 | 7.04 |  |  |  |  |  |  |
| Butanoate metabolism | Yak | 5.11 | 5.22 | 6.80 | 6.63 | 0.027 | 0.896 | 0.026 | 0.644 | 0.011 | 0.240 |
|  | Cattle | 6.15 | 6.50 | 6.60 | 6.63 |  |  |  |  |  |  |
| Pentose and glucuronate interconversions | Yak | 4.44 | 4.15 | 5.06 | 5.53 | 0.036 | 0.117 | 0.175 | 0.922 | 0.377 | 0.052 |
|  | Cattle | 5.45 | 5.37 | 5.35 | 5.89 |  |  |  |  |  |  |
| **Energy Metabolism** | | | | | | | | | | | |
| Methane metabolism | Yak | 10.1 | 10.7 | 12.9 | 12.8 | 0.04 | 0.464 | 0.010 | 0.404 | 0.001 | 0.430 |
|  | Cattle | 12.2 | 12.6 | 12.9 | 13.5 |  |  |  |  |  |  |
| Oxidative phosphorylation | Yak | 11.1 | 11.3 | 12.1 | 11.2 | 0.07 | 0.161 | <0.01 | 0.661 | <0.001 | 0.143 |
|  | Cattle | 12.8 | 12.6 | 12.2 | 10.8 |  |  |  |  |  |  |
| Carbon fixation pathways in prokaryotes | Yak | 9.68 | 9.56 | 11.2 | 10.6 | 0.036 | 0.287 | 0.088 | 0.313 | 0.016 | 0.457 |
|  | Cattle | 11.1 | 11.2 | 11.1 | 10.8 |  |  |  |  |  |  |
| Nitrogen metabolism | Yak | 6.11 | 5.88 | 7.31 | 6.93 | 0.025 | 0.665 | 0.037 | 0.348 | 0.026 | 0.200 |
|  | Cattle | 7.26 | 7.31 | 7.27 | 6.60 |  |  |  |  |  |  |
| Carbon fixation in photosynthetic organisms | Yak | 5.55 | 5.49 | 6.57 | 6.86 | 0.021 | 0.862 | 0.327 | 0.987 | 0.348 | 0.116 |
|  | Cattle | 6.70 | 6.52 | 6.54 | 6.85 |  |  |  |  |  |  |
| **Amino Acid Metabolism** | | | | | | | | | | | |
| Arginine and proline metabolism | Yak | 10.9 | 10.6 | 13.0 | 12.5 | 0.03 | 0.351 | 0.324 | 0.185 | 0.133 | 0.830 |
|  | Cattle | 12.8 | 13.0 | 13.0 | 12.8 |  |  |  |  |  |  |
| Alanine, aspartate and glutamate metabolism | Yak | 10.1 | 9.76 | 11.5 | 11.4 | 0.03 | 0.800 | 0.016 | 0.631 | <0.01 | 0.411 |
|  | Cattle | 11.9 | 11.8 | 11.6 | 11.4 |  |  |  |  |  |  |
| Cysteine and methionine metabolism | Yak | 8.23 | 8.11 | 9.89 | 9.72 | 0.016 | 0.016 | 0.918 | 0.490 | 0.813 | 0.832 |
|  | Cattle | 9.91 | 10.1 | 10.1 | 10.0 |  |  |  |  |  |  |
| Phenylalanine, tyrosine and tryptophan biosynthesis | Yak | 8.19 | 7.96 | 9.43 | 9.31 | 0.027 | 0.226 | 0.528 | 0.075 | 0.553 | 0.188 |
|  | Cattle | 9.60 | 9.59 | 9.59 | 9.95 |  |  |  |  |  |  |
| Glycine, serine and threonine metabolism | Yak | 8.10 | 7.75 | 8.97 | 8.52 | 0.032 | 0.925 | <0.01 | 0.646 | <0.001 | 0.934 |
|  | Cattle | 9.43 | 9.29 | 9.04 | 8.70 |  |  |  |  |  |  |
| Lysine biosynthesis | Yak | 7.07 | 7.09 | 8.50 | 8.20 | `0.022 | 0.259 | 0.995 | 0.144 | 0.993 | 0.788 |
|  | Cattle | 8.41 | 8.49 | 8.54 | 8.70 |  |  |  |  |  |  |
| Valine, leucine and isoleucine biosynthesis | Yak | 7.68 | 7.83 | 7.96 | 8.23 | 0.023 | 0.040 | <0.01 | 0.114 | 0.042 | 0.937 |
|  | Cattle | 6.50 | 6.52 | 7.97 | 7.70 |  |  |  |  |  |  |
| Histidine metabolism | Yak | 5.80 | 5.93 | 6.98 | 6.73 | 0.016 | 0.137 | 0.162 | 0.401 | 0.256 | 0.061 |
|  | Cattle | 6.86 | 6.85 | 6.82 | 6.77 |  |  |  |  |  |  |
| **Metabolism Of Cofactors and Vitamins** | | | | | | | | | | | |
| One carbon pool by folate | Yak | 6.59 | 6.36 | 7.06 | 6.94 | 0.032 | 0.603 | <0.01 | 0.498 | <0.001 | 0.908 |
|  | Cattle | 7.63 | 7.53 | 7.31 | 6.79 |  |  |  |  |  |  |
| Pantothenate and CoA biosynthesis | Yak | 6.12 | 6.00 | 6.96 | 6.69 | 0.019 | 0.775 | <0.01 | 0.489 | <0.001 | 0.184 |
|  | Cattle | 7.17 | 7.20 | 7.13 | 6.62 |  |  |  |  |  |  |
| Porphyrin and chlorophyll metabolism | Yak | 5.74 | 5.32 | 7.53 | 6.49 | 0.069 | 0.701 | 0.110 | 0.519 | 0.307 | 0.210 |
|  | Cattle | 6.97 | 7.28 | 7.36 | 6.22 |  |  |  |  |  |  |
| **Nucleotide Metabolism** | | | | | | | | | | | |
| Purine metabolism | Yak | 19.6 | 19.3 | 22.8 | 23.3 | 0.04 | 0.699 | 0.143 | 0.972 | 0.233 | 0.068 |
|  | Cattle | 23.4 | 23.1 | 22.8 | 23.3 |  |  |  |  |  |  |
| Pyrimidine metabolism | Yak | 17.5 | 17.3 | 20.2 | 20.2 | 0.03 | 0.612 | 0.031 | 0.520 | <0.01 | 0.481 |
|  | Cattle | 20.6 | 20.5 | 20.3 | 20.3 |  |  |  |  |  |  |
| **Glycan Biosynthesis and Metabolism** | | | | | | | | | | | |
| Peptidoglycan biosynthesis | Yak | 7.45 | 7.21 | 8.72 | 8.75 | 0.022 | 0.084 | 0.289 | 0.540 | 0.666 | 0.066 |
|  | Cattle | 9.00 | 8.80 | 8.81 | 9.15 |  |  |  |  |  |  |
| **Metabolism Of Terpenoids and Polyketides** | | | | | | | | | | | |
| Terpenoid backbone biosynthesis | Yak | 5.50 | 5.38 | 6.40 | 6.37 | 0.012 | 0.315 | 0.047 | 0.813 | <0.01 | 0.827 |
|  | Cattle | 6.51 | 6.46 | 6.39 | 6.26 |  |  |  |  |  |  |
| **Genetic Information Processing** | | | | | | | | | | | |
| **Translation** | | | | | | | | | | | |
| Ribosome | Yak | 23.1 | 23.1 | 26.8 | 27.1 | 0.05 | 0.260 | 0.207 | 0.592 | 0.101 | 0.498 |
|  | Cattle | 27.3 | 27.1 | 26.8 | 27.0 |  |  |  |  |  |  |
| Aminoacyl-tRNA  biosynthesis | Yak | 10.4 | 10.6 | 12.8 | 13.0 | 0.02 | 0.446 | <0.01 | 0.631 | <0.01 | 0.606 |
|  | Cattle | 12.5 | 12.5 | 12.6 | 13.0 |  |  |  |  |  |  |
| **Replication and Repair** | | | | | | | | | | | |
| Homologous  recombination | Yak | 8.92 | 8.90 | 10.5 | 10.6 | 0.014 | 0.957 | 0.209 | 0.116 | 0.399 | 0.125 |
|  | Cattle | 10.6 | 10.6 | 10.6 | 10.9 |  |  |  |  |  |  |
| Mismatch repair | Yak | 7.57 | 7.48 | 8.97 | 8.84 | 0.012 | 0.063 | 0.793 | 0.119 | 0.700 | 0.359 |
|  | Cattle | 9.06 | 9.03 | 9.05 | 9.18 |  |  |  |  |  |  |
| DNA replication | Yak | 6.29 | 6.25 | 7.35 | 7.32 | 0.011 | 0.476 | 0.224 | 0.292 | 0.095 | 0.424 |
|  | Cattle | 7.44 | 7.40 | 7.33 | 7.42 |  |  |  |  |  |  |
| **Folding, Sorting and Degradation** | | | | | | | | | |  |  |
| Protein export | Yak | 5.71 | 5.72 | 6.57 | 6.69 | 0.016 | 0.162 | 0.184 | 0.417 | 0.235 | 0.264 |
|  | Cattle | 6.67 | 6.61 | 6.54 | 6.69 |  |  |  |  |  |  |
| **Environmental Information Processing** | | | | | | | | | | | |
| **Membrane Transport** | | | | | | | | | | | |
| ABC transporters | Yak | 17.4 | 19.7 | 27.9 | 30.2 | 0.27 | 0.892 | 0.015 | 0.185 | <0.01 | 0.511 |
|  | Cattle | 23.8 | 25.7 | 27.0 | 26.6 |  |  |  |  |  |  |
| Bacterial secretion system | Yak | 5.15 | 5.11 | 6.08 | 6.18 | 0.021 | 0.967 | 0.170 | 0.120 | 0.160 | 0.091 |
|  | Cattle | 5.99 | 5.98 | 6.03 | 6.56 |  |  |  |  |  |  |
| **Signal Transduction** | | | | | | | | | | | |
| Two-component system | Yak | 9.26 | 9.46 | 13.0 | 12.5 | 0.12 | 0.220 | 0.208 | 0.711 | 0.087 | 0.573 |
|  | Cattle | 12.2 | 12.6 | 13.1 | 12.6 |  |  |  |  |  |  |
| **Cellular Processes** | | | | | | | | | | | |
| **Cell Growth and Death** | | | | | | | | | | | |
| Cell cycle - Caulobacter | Yak | 4.86 | 4.75 | 5.50 | 5.43 | 0.014 | 0.436 | <0.01 | 0.762 | <0.001 | 0.940 |
|  | Cattle | 5.74 | 5.63 | 5.56 | 5.35 |  |  |  |  |  |  |

SEM = standard error of the means; ME = metabolizable energy.

^1^ S = species; E = dietary energy level; E-L = Linear effect of dietary energy levels; E-Q = Quadratic effect of dietary energy levels.
